# The clinical and microbiological efficacy of a zinc-citrate/hydroxyapatite/potassium-citrate containing toothpaste: a double-blind randomized controlled clinical trial

**DOI:** 10.1007/s00784-024-06052-z

**Published:** 2024-11-22

**Authors:** Uros Josic, Tatjana Maravic, Claudia Mazzitelli, Lorenzo Rinaldi, Carlo D’Alessandro, Diego D’Urso, Gerardo Pellegrino, Annalisa Mazzoni, Lorenzo Breschi

**Affiliations:** Department for Biomedical and Neuromotor Sciences, DIBINEM, University of Bologna, Via San Vitale 59, 40125 Bologna, Italy

**Keywords:** Antimicrobial, Dentin sensitivity, Randomized clinical trial, Toothpaste, Fluoride, Zinc citrate, Hydroxyapatite

## Abstract

**Objectives:**

To evaluate the antibacterial efficacy of two fluoride-containing (1450 ppm F) toothpastes with or without zinc-citrate (ZCT), hydroxyapatite (HAP) and potassium-citrate (KCit); to assess and compare their clinical effects in terms of tooth sensitivity, plaque accumulation and gingivitis, as well as patients’ satisfaction.

**Materials and methods:**

Healthy, adult patients were selected and randomly assigned to two groups (*n* = 50): Experimental: ZCT-, HAP-, KCit- and fluoride-containing toothpaste; Control: fluoride-containing toothpaste. Salivary counts of *Streptococcus mutans (S. mutans)*, plaque and gingival index, as well as clinically diagnosed sensitivity were recorded at baseline, and after 4 weeks. A custom-made questionnaire was used to assess patients’ self-reported sensitivity (baseline and after 4 weeks) and overall satisfaction with the tested toothpastes. Data were statistically analyzed (α = 0.05).

**Results:**

After 4 weeks, a statistically significant salivary reduction of *S. mutans* was observed in both groups (*p* = 0.001). Furthermore, the percentage of *S. mutans* decrease was significantly higher in Experimental group (*p* = 0.014). There were no statistically significant differences between the groups in terms of plaque and gingival index (*p* > 0.05). After 4 weeks, the self-reported tooth sensitivity was lower in Experimental group (*p* < 0.001).

**Conclusions:**

Both toothpastes showed good antimicrobial effect after 4 weeks; however, the toothpaste containing ZCT, HAP, KCit and fluoride was found to be more effective in reducing the salivary counts of *S. mutans* than the product containing fluoride alone.

**Clinical relevance:**

Toothpaste containing ZCT, HAP, KCit and fluoride can be recommended for patients at risk for developing caries and may also be beneficial for individuals experiencing dental sensitivity.

**Supplementary information:**

The online version contains supplementary material available at 10.1007/s00784-024-06052-z.

## Introduction

Dental caries remains a large burden for health-care systems around the world as it can affect children [1], middle-aged and elderly populations [2]. A recent systematic review highlighted the potential influence of genetic factors on caries development [3], and it is well established that socio-demographic inequality plays a major role in caries distribution [4]. Furthermore, emerging evidence suggests that it can be associated with systemic diseases [5] and can be an important issue in patients who received an organ transplant [6]. Secondary caries remains the main reason for failure and reduced lifespan of resin-composite restorations placed on posterior teeth [7]. The etiology of dental caries has been widely investigated in the past and, nowadays, most of the authors agree that, among many factors, the most significant one is the presence of *Streptococcus mutans (S. mutans)* which can colonize tooth surfaces and form cariogenic biofilms. The virulence of *S. mutans* biofilms is attributed to acid production, the capacity to resist in harsh environment and the ability to produce extracellular polymers [8].

Other common conditions affecting the oral health are gingivitis and dentin hypersensitivity (DH). The first one, caused by inadequate biofilm control, represents a direct risk factor for the development of periodontal disease and loss of teeth [9]. Additionally, severe forms of periodontal diseases are associated with diabetes, cardiovascular diseases, pregnancy complications, rheumatoid arthritis and even cognitive pathologies such as Alzheimer’s disease [10]. On the other hand, DH has no severe effect on systemic health, but can have a negative impact on oral health-related quality of life as it can impair daily activities such as eating, speaking, drinking and toothbrushing [11]. The average prevalence of DH calculated from worldwide cross-sectional studies is reported to be 33.5%; interestingly, only 22% of the studies that can be found in the literature employed both self-reported and clinically-assessed DH to diagnose it [12].

Adequate control and removal of dental plaque by means of everyday oral hygiene effectively prevents the development of caries and gingivitis. The probability of developing a caries lesion is higher in non-frequent brushers, and this is particularly evident in deciduous dentition [13]. In order to further enhance the effect of mechanical brushing, fluorides (F) have been added to dentifrices and, in concentration between 1000 and 1500 ppm F, are by far the most important and effective way of preventing tooth decay [14]. The effect is ascribed to the local action of fluorides on tooth/biofilm surface, as it prevents enamel solubility, enhances remineralization [15] and may have inhibitory effect on virulence factors and composition of *S. mutans* biofilm [16]. Other ingredients, such as hydroxyapatite (HAP), have recently been added to the composition of contemporary dentifrices; many in vitro studies [17] confirmed HAP’s remineralizing and caries-arrest potential, and it is also believed that it can reduce DH and biofilm formation. These theories still need to be strengthen by clinical evidence [18]. Similarly, the addition of zinc-citrate (ZCT) in F-containing dentifrices was found to be clinically more efficient in reducing the number of anaerobic bacteria and streptococci compared to the F-alone toothpaste after 14 days of use [19]. The inclusion of potassium-citrate (KCit) in formulation of dentifrices should help reduce DH, as these salts decrease the excitability of the dentinal nerves by changing their membrane potential [20].

Introducing a multi-component dentifrice that combines these various active ingredients (such as HAP, ZCT, and KCit) with F may offer a synergistic approach to oral health. This multi-component formulation could potentially address multiple oral health issues simultaneously, including caries prevention, biofilm control, reduction of hypersensitivity, and improvement of periodontal health. Therefore, the primary aim of this double-blind randomized controlled clinical trial (RCT) was to compare the antimicrobial efficacy, in particular its ability to reduce salivary counts of *S. mutans*, of a recently introduced ZCT-, HAP- and KCit-, F- containing toothpaste to a dentifrice containing F. The secondary aims were to investigate the differences in plaque and gingival indices, DH as well as patients’ satisfaction after a 4-week use of the tested dentifrices. The tested null hypotheses were that: (1) no differences would be found in terms of reduction of salivary counts of *S. mutans* between the two dentifrices; (2) the type of dentifrice would have no influence on plaque and gingival indexes, and (3) there would be no differences regarding clinically-diagnosed and self-reported DH between the two groups using different dentifrices.

## Materials and methods

### Ethical approval and study registration

This was a single-site, double-blind RCT that was conducted in the Dental Clinic of Bologna University (DIBINEM), Bologna, Italy in the period from November 2022 until July 2023. Before initiating the trial, the study protocol was reviewed and approved by the University Ethics Committee (793/2022/SPER/AUSLBO) and the trial was registered at Clinicaltrials.gov (NCT05569850). The trial was in accordance with Helsinki Declaration of Human Rights [21] and followed the CONSORT statement [22]. The tested dentifrices included a newly introduced ZCT-, HAP- and KCit- 1450 ppm sodium monofluorophosphate containing (Experimental group; Mentadent PROTECT + Carie, Mentadent, Unilever, Milan, Italy) and 1450 ppm NaF containing (Control group; AZ Multi-Protezione Scudo Protettivo Famiglia) toothpastes. Table 1 shows the list composition of both tested products.

**Table 1:** List of ingredients in the tested toothpastes

Product’s commercial name	List of ingredients
Mentadent PROTECT+ Carie, Mentadent, Unilever, Milan, Italy	Water, Hydrogenated Starch Hydrolysate, Hydrated Silica, Potassium Citrate, Hydroxyapatite, Zinc Citrate, PEG-32, Sodium Lauryl Sulfate, Flavor, Sodium Monofluorophosphate, Trisodium Phosphate, Cellulose Gum, Sodium Hydroxide, Sodium Saccharin, Limonene. Sodium Monofluorophosphate (1450 ppm F)
AZ MULTIPROTEZIONE Scudo Protettivo Famiglia	Water, Hydrated Silica, Sorbitol, Sodium Lauryl Sulfate, Cellulose Gum, Flavor, Sodium Fluoride, Sodium Saccharin, Carbomer, CI 77,891 (Titanium Dioxide), Trisodium Phosphate, Limonene, Polysorbate 80, CI 74,260 (Green Colorant). Sodium Fluoride (1450 ppm F)

### Sample size, recruitment and eligibility criteria

The number of patients was determined based on the power analysis performed using data from a previously published study [23] in order to assure a power of 80% for finding the statistically significant differences for the study’s primary outcome (change in *S. mutans* salivary counts) given the standard value of type I errors (0.05). Based on the outcomes of the power calculation (effect size f = 0.2291667, at the level of α 0.05, and 1-β prob 0.80), the determined sample size was 92 patients. Considering the probability of loss to follow-up 10% of the minimum size per group was added, leading to the final sample size of 100 patients.

**Fig. 1:**
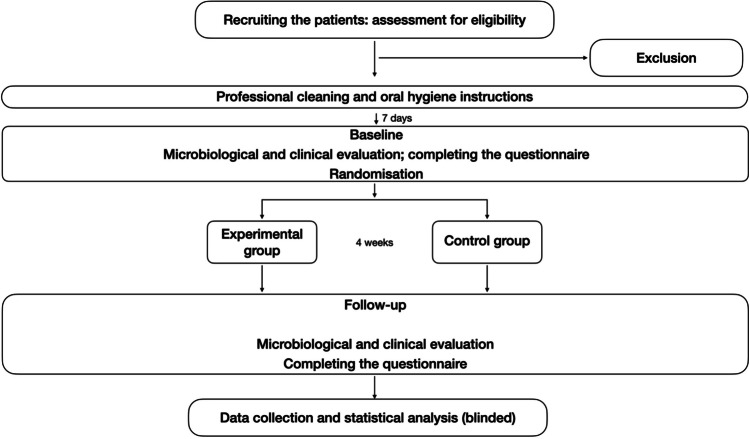
Study flowchart

Potential patients were recruited through local advertisement or within Dental Clinic (DIBINEM) at Bologna University, Bologna, Italy during routine dental check-ups. Study details were explained to the potential participants and only those who voluntarily signed the informed consent were scheduled for the first visit (screening, Fig. 1). During the screening phase, one dentist evaluated the eligibility of the patients in the study based on the following inclusion criteria: male and female patients aged 18–50 with minimum of 20 natural teeth in stable occlusion and good oral hygiene level (bleeding on probing not exceeding 20%, no advanced periodontal disease, absence of active caries lesions, pulpitis), patients with good general health (no systemic diseases reported in the medical anamnesis), and subjects with good language comprehension. Exclusion criteria were tooth anomalies (i.e. amelogenesis imperfecta, dentinogenesis imperfecta etc.), intrinsic stain (i.e. fluorosis, molar incisors hypomineralization [MIH]), active caries lesions, advanced periodontal disease, smokers, presence of orthodontic devices, pregnancy, lactation, use of antibiotics in the last 3 months, use of antibacterial mouthrinses in the last 3 months, reported allergies, drug and alcohol addiction.

### Study procedures

One week before the beginning of the trial, all patients received sub- and supra-gingival full-mouth prophylaxis [24] during which a fluoride-free paste (Nupro, Dentsply Sirona) was used. At the same appointment, patients were given detailed oral hygiene instructions by the dental hygienist and a brief explanatory video was shared via messaging applications (i.e., WhatsApp) to serve as a reminder for good habits consulting during the study period. The patients were scheduled to come back after 7 days (baseline visit) and were asked not to brush or eat at least 5 h before the appointment.

During the baseline visit, all patients completed a custom-made questionnaire (supplementary file) containing questions regarding DH (“self-reported” sensitivity), their perception of tooth color and overall perception of the toothpaste they were using at the time. Subsequently, they were asked to collect 2 ml of unstimulated saliva in sterile tubes (Greiner centrifuge tube, Sigma-Aldrich, St Louis, MO, USA). Saliva was immediately processed for microbiological analysis as thereafter described. Upon saliva collection, a sensitivity test was conducted by applying a tactile and air-stimulus (duration: 2 s, distance from the tooth surface: 1 cm) [25] test to all teeth of the four quadrants. The subjects’ response was recorded on a 4-point Schiff Sensitivity Scale [26] and visual-analogue scale [25], respectively. Additionally, plaque index (Modified Quigley and Hein) [27] was scored on a 6 point scale at 3 buccal and 3 lingual sites per tooth after it had been disclosed with a plaque detector (Biofilm Disclosure, EMS Dental, Nyon, Switzerland); gingival index according to Löe-Silness [28] was also recorded, as well as bleeding on probing index. Patients’ tooth color was determined under daily light using the Vita classical shade guide [29]. Finally, at the end of the baseline visit, the participants were provided with the same soft-bristled toothbrush (Mentadent P, Mentadent, Unilever) and randomly assigned into one of the two groups (*n* = 50) according to the dentifrice used. An online software (https://www.sealedenvelope.com) was utilized for randomization purposes by a staff member unaware of the study protocol. In order to enable complete blinding of the participants and dental examiner to the group assignment, the two dentifrices were distributed in identical plain white tubes provided with only letters A or B, with no other visible marks. The patient’s code and dentifrice assigned (A or B) were documented in the chart for later reference. From the baseline visit, the subjects had to restrain from using any other products or means of oral hygiene except those provided and were invited to follow the domiciliary oral hygiene instructions as previously described. In case of any adverse events were to occur, the subjects were advised to discontinue the use of the toothpaste and were withdrawn from the study. The 4 weeks follow-up visit was fixed (recall).

After 4 weeks (recall), the patients returned to the study site for a final visit after having abstained from brushing or eating at least 5 h before the visit. During this visit, the patients were asked to fill in the questionnaire (supplementary file), while the saliva collection and all clinical procedures described above were repeated by the same dentist [30].

### Microbiological procedures

The saliva collected during the baseline and recall visits was processed for microbiological analysis within 3 h of sample acquisition [31]. Briefly, 0.1 ml of saliva was diluted in 9.9 ml of sterile phosphate-buffered saline solution until 10^−8^ was reached. Subsequently, the diluted samples were dispersed on Mitis Salivarius Selective Agar (MSB, Microbiol Diagnostici, UTA, CA, Italy) [32] and the plates were incubated at 37^o^C for the next 48–72 h. The bacterial colonies were identified by morphology [33] and counted by one trained investigator, blinded to the groups. *S. mutans* colonies (CFU) were multiplied by their respective dilution ratio to calculate the number of colonies per one milliliter (CFU/mL) of each subject’s saliva sample. In order to perform statistical analysis, a base-10 logarithm transformation was applied to the calculated CFU/ml values [23, 34, 35].

### Statistical analysis

After checking the normality (Kolmogorov–Smirnov) and the homoscedasticity (modified Levene’s test) of the data, the Two Way Repeated Measures ANOVA (One Factor Repetition) followed by Bonferroni post-hoc were run to investigate the effect of the tested dentifrices on salivary counts of *S. mutans*, as well as plaque and gingival indexes. Since the data retrived from questionnares where not normally distributed (Kolmogorov–Smirnov, *p* < 0.05), the non-parametric Mann-Whitney U-test was run. All analyses were performed by a statistician blinded to the groups using SigmaPlot 14.0 (Systat Software, Chicago, IL, USA). In all tests, the significance level was set at α = 0.05.

## Results

**Fig. 2:**
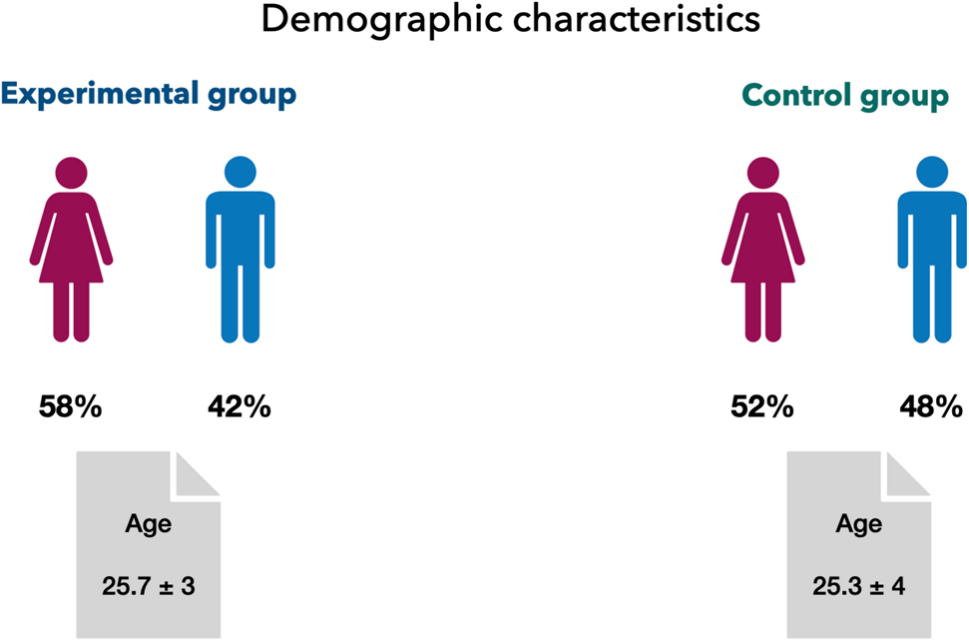
Demographic characteristics of patients enrolled in the study

**Fig. 3:**
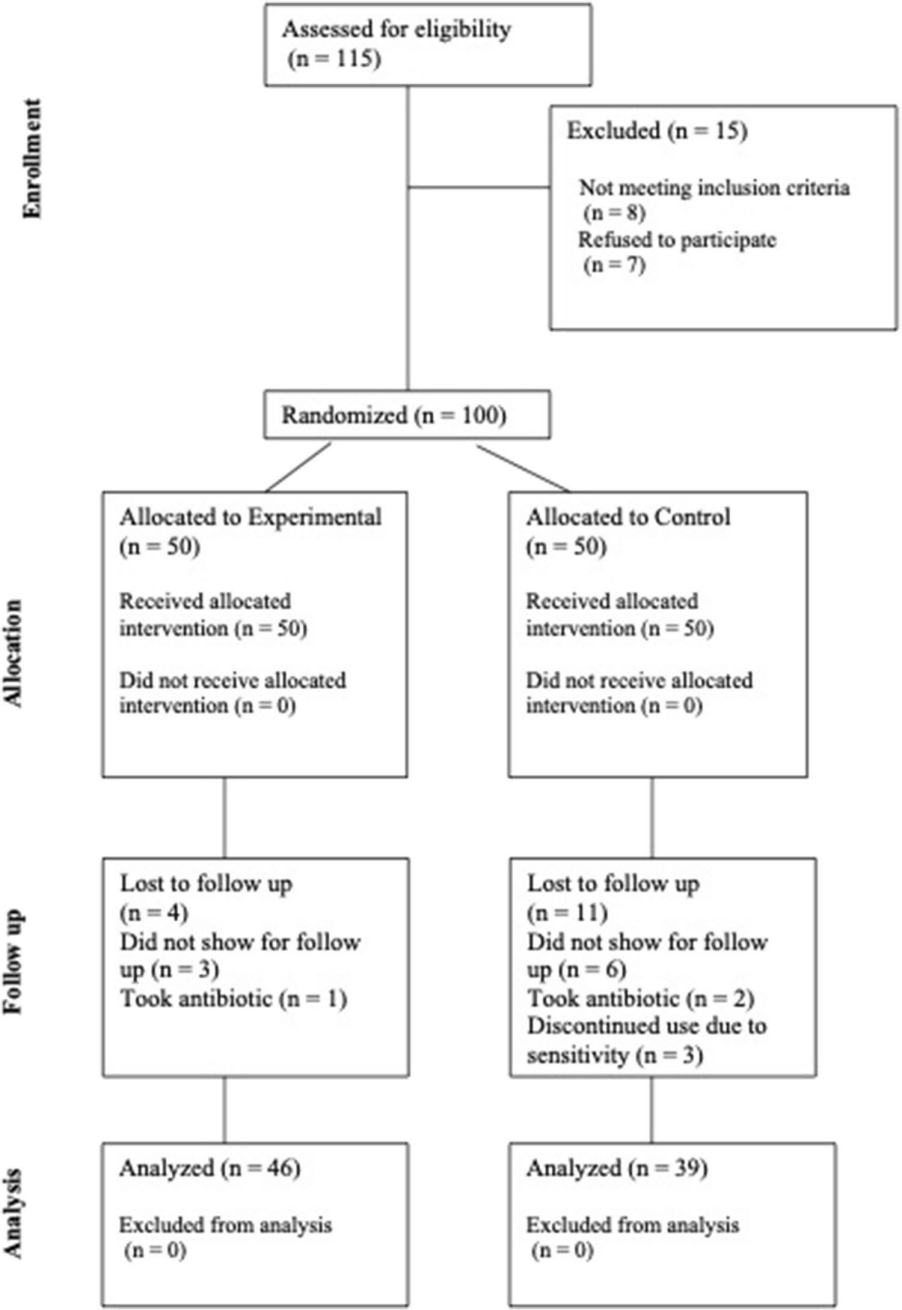
CONSORT 2010 flow diagram

A total of 100 patients were enrolled in this double-blind RCT and were randomly assigned into Control (*n* = 50; 1450 ppm F-containing toothpaste) and Experimental group (*n* = 50; ZCT-, HAP-, KCit- 1450 ppm F-containing toothpaste). The demographic characteristics of the subjects included in this RCT are shown in Fig. 2. Age and sex distribution were similar across the two study groups. At the end of the follow-up period (recall), 15 participants (4 in Experimental group and 11 in Control group) were lost due to the reasons listed in CONSORT flow-chart (Fig. 3). Common reasons for drop-out were failure to return for the recall visit despite the authors’ attempt to contact the patients or antibiotics administration due to infections non-related to oral cavity. No serious adverse reactions such as allergies occurred during the trial. Three patients in Control group discontinued the use of their dentifrices because they experienced increased DH. This symptom, however, disappeared once the use of the product was interrupted.

**Fig. 4:**
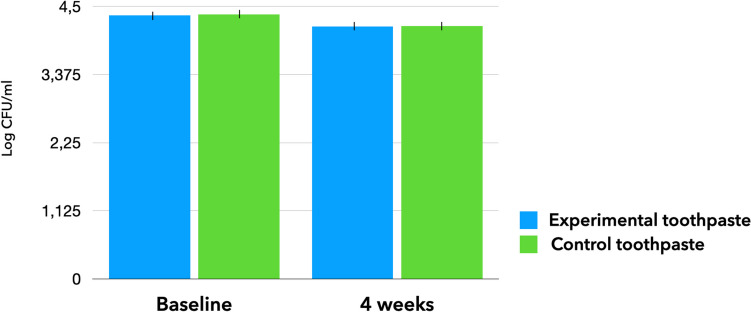
Mean amount of change in the logarithm of colony-forming units per milliliter (log CFU/mL) of *S. mutans* in salivary samples at baseline and at the end of the follow-up (4 weeks)

**Fig. 5:**
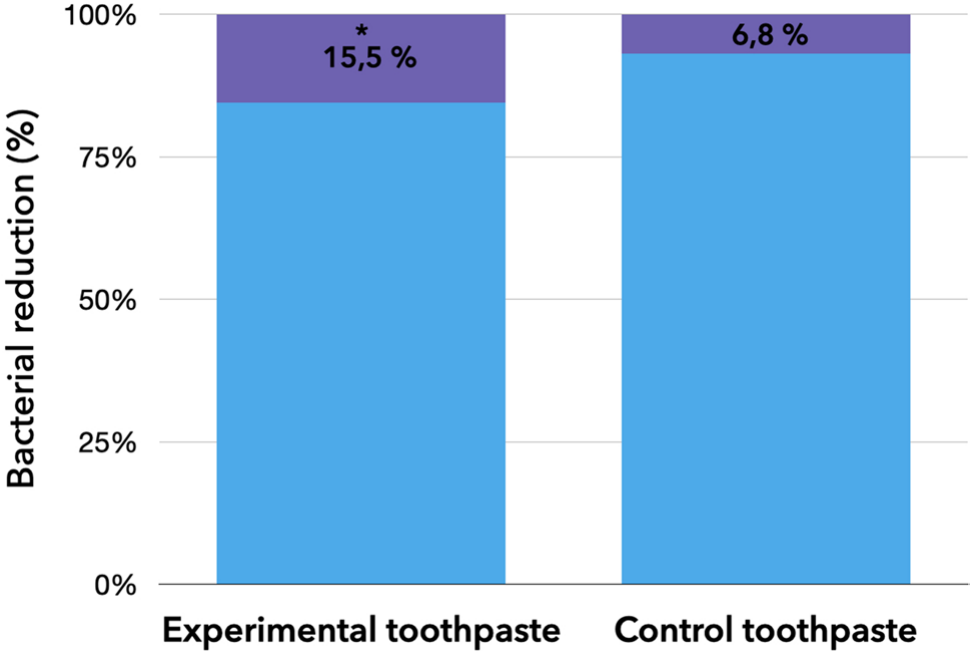
The percentage of *S. mutans* reduction (shown as purple) in salivary samples of patients in experimental and control toothpaste groups. In case of statistically significant differences between the groups (*p* < 0.05), an asterisk was placed next to the significantly higher value for the outcome

Figure 4 shows salivary counts of *S. mutans* expressed as log CFU/ml for the tested dentifrices, at baseline and after 4 weeks (recall). The regular use of both dentifrices over the studied period significantly decreased salivary counts of *S. mutans* compared to the baseline, with statistical significance (*p* = 0.001). Furthermore, the percentage of *S. mutans* reduction was significantly higher (*p* = 0.014) in Experimental than in Control group (15.5% vs. 6.8%, respectively, Fig. 5). No statistically significant differences were observed for other clinically assessed parameters (*p* > 0.05, Supplementary file).

The data retrieved from questionaries revealed that, after 4 weeks of dentifrice use, a higher percentage (23%) of patients in Control group experienced stronger DH compared to Experimental group (10%; *p* < 0.001). Conversely, a higher percentage (34% vs. 7%) of patients in Experimental group reported the sensation of “dry mouth” after toothbrushing (*p* = 0.003). Additionally, a greater percentage of participants in Control compared to Experimental group (93% vs. 63%, respectively) expressed satisfaction with the dentifrice’s flavor (*p* < 0.001), indicated intentions to continue using it in future (66% vs. 44%, respectively; *p* = 0.023) and reported overall positive evaluation of the product used (48% vs. 36%, respectively; *p* = 0.015). No difference in tooth color was found between the groups regarding participants’ perception (*p* > 0.05).

## Discussion

Caries and periodontal disease continue to be the leading factors responsible for tooth loss in adult population [36]. Although their etiology has been elucidated and it is generally accepted that rigorous biofilm control is fundamental to prevent their development [37], achieving optimal level of oral hygiene that would reduce bacterial load in oral cavity is still challenging. For this purpose, many efforts have been put into developing dentifrices which, besides fluorides, contain ingredients that could provide a more comprehensive protection, broader range of antibacterial effect, favor remineralization further and help reduce DH [38]. Consequently, in this RCT, we aimed to clinically and microbiologically assess the effect of ZCT, HAP and KCit addition to a sodium monofluorophosphate toothpaste and compare it to a control toothpaste containing the same dose of F.

After 4 weeks of regular brushing with the tested dentifrices, the salivary count of *S. mutans* was significantly reduced in both groups compared to the baseline. Furthermore, the percentage of bacterial reduction was higher in Experimental group at the end of the follow-up, which led to the rejection of the first null hypothesis.

The reduction of bacterial load in both groups that was observed in this study can possibly be explained by the Hawthorne effect [39]: patients enrolled in a clinical trial frequently appear to improve their oral hygiene habits and compliance due to the nature of the study which implies detailed examination and documentation of each participant during regular check-ups. The Hawthorne further states that subjects who are aware that they received a potentially less effective product may not comply to the entire study protocol like patients who were given a product that is expected to have superior properties [38]. By conducting a double-blind RCT in which both patients and evaluators were unaware of the group assignment (in this case, the type of dentifrice used during the trial), we were able to eliminate the second issue related to the Hawthorne effect, assuring good compliance among all patients which was confirmed by visual inspection of the toothbrushes and dentifrices after 4 weeks of daily use. In comparison to the baseline, lower salivary colonization of *S. mutans* observed at the end of this study can be attributed to rigorous oral hygiene regime maintained by patients in both groups throughout the entire duration of this RCT. This agrees with previously published research [23, 40–42].

The addition of F into toothpaste composition has brought major benefits for caries prevention. Compared to non-F dentifrices, F products show superior protection against caries and should be advised for every day at-home oral hygiene, taking into consideration patients’ age as to avoid the risk of fluorosis development [15]. Instead of using a placebo, F-free dentifrice which, up to a certain extent, would have left the patients unprotected from caries during the course of our study [43, 44], in this RCT, a commercially available NaF-based dentifrice was used as control with the same concentration of F (1450 ppm) as in the recently-introduced ZCT-, HAP- and KCit-containing dentifrice.

A greater decrease of *S. mutans* salivary counts which we observed in the Experimental group can most probably be explained by the addition of ZCT to the formula of the novel dentifrice. The general antibacterial activity of zinc is well known [45, 46], and it is usually incorporated in dentifrices with the principal aim of preventing halitosis [47]. It has been reported that patients who used a 0.5% ZCT-containing dentifrice had higher concentrations of zinc ions in their unstimulated saliva, for at least 4 h after brushing [48]. These zinc ions are considered important for inhibiting glucose metabolism of *S. mutans* [49]. The results of our study confirm previous findings which stated that ZCT-containing F toothpaste is more efficient than the control dentifrice containing only F in reducing the salivary load of streptococci after 2 weeks of brushing [19]. Similarly, Hu et al. reported that ZCT-dentifrice significantly reduced the amount of anaerobic bacteria found in dental biofilm, tongue, cheek and saliva in patients who regularly brushed their teeth for 2 weeks [50]. Consistent with the mentioned studies, we observed that the addition of ZCT to a F-containing dentifrice could be clinically beneficial for reducing the salivary *S. mutans* load and that the synergistic effect of fluoride and ZCT on reducing glycolysis and acid tolerance of *S. mutans* [51] was not influenced by the incorporation of additional therapeutical components such as HAP and KCit in the formulation of the tested dentifrice.

Moreover, it was previously reported that nano-HAP dentifrice can reduce in vitro *S. mutans* biofilm formation on enamel and resin-composite materials [52] and that the combination of zinc and HAP can inhibit *S. mutans* development [53]. Accordingly, the presence of HAP in the Experimental group may have contributed to better antibacterial efficacy.

Recording plaque and periodontal indices represent a useful and unreplaceable tool in monitoring patients’ compliance and eventual changes of periodontal health in clinical trials [54, 55]. Carefully measured and well documented indices allow researchers to evaluate the efficacy of a surgical or conservative therapy, as well as the effectiveness of using novel cosmetical products such as dentifrices. In this study, we observed no differences for the evaluated indices, between baseline and the end of the trial - hence the second null hypothesis could not be rejected.

Previously, Daly et al. (2019) found an improvement in plaque and gingival score in patients who used a F dentifrice containing enzymes and proteins for 3 months, compared to the F-alone dentifrice [56]. Similarly, Lorenz et al. (2018) reported that stannous chloride toothpaste that contained F was more efficient in reducing scores for plaque and gingival index after 3 and 12 weeks of brushing [57]. A recent systematic review with meta-analysis confirmed that toothbrushing with a standard F dentifrice does not provide an added effect for the mechanical removal of dental plaque. However, dentifrices containing chemical agents such triclosan or stannous F offer benefits with respect to gingival health and control of dental plaque compared to F-only dentifrices [58]. The addition of ZCT to a F dentifrice also showed higher reduction of plaque and gingival parameters after 1 and 3 months of clinical use compared to F dentifrice [19, 59]. The absence of statistically significant differences related to indices in our RCT may be explained by the study design: one week prior to the beginning of the study, oral prophylaxis was performed by an oral hygienist and detailed oral hygiene instructions were provided to the patients via in-office demonstration and a video registration which could have been re-watched. This led to inclusion of patients who had excellent baseline index scores (most of them were slightly above 0), whose values were maintained throughout the study.

DH is characterized by short, sharp pain when teeth are exposed to stimulus. The etiology of DH is diverse and many theories have been proposed regarding its mechanism including the classic hydrodynamic theory, direct innervation of dentinal tubules, neuroplasticity and sensitization of nociceptors, odontoblasts serving as sensory receptors and algoneurons [60]. The introduction of desensitizing agents into toothpastes is a well-established and promising approach in reducing DH. For this purpose, potassium and strontium salts are commonly used as they can enter dentinal tubules, reach the nerves and decrease their excitability by altering membrane potential [20]. A more recent approach in controlling DH is the incorporation of HAP which can occlude dentinal tubules at microscopic level [61]. The addition of HAP and KCit salts into the formulation of the novel dentifrice may be responsible for the lower incidence of DH in Experimental group compared to Control group. Our observation is in line with a previous study that found beneficial effect of a zinc–carbonate HAP dentifrice on self-reported DH after 4 weeks of daily brushing [62]. Similarly, the incidence of self-reported DH after 2- and 4-weeks was also minor in patients who used nano-HAP dentifrice compared to F-alone toothpaste [63]. The results of our study support the theory that the combination of more active ingredients with different mode of action can potentially increase their efficacy in reducing DH [64]. It is, however, important to highlight that we observed improvement only in self-reported, whereas no differences were found in clinically-assessed DH. This is most likely due to the fact that some subjects may report any form of dental pain or discomfort as sensitivity in daily life [65]. Indeed, although more subjective, DH is usually more frequently encountered when employing a questionnaire (self-reported DH) as compared to in-office provoked stimuli within non-carious cervical lesions restored with resin-composite materials [66, 67].

Finally, some considerations related to this study should be mentioned. Our primary aim was to evaluate the efficacy in reducing salivary counts of *S. mutans*. In future, it would be of interest to consider the effect of the tested toothpaste on biofilm masses located on tooth and oral mucosa surfaces, as well as to investigate its influence on other bacterial species in longer-term studies. Recruiting and distributing a homogenous patient sample in terms of initially reported DH would be desirable as to further elucidate the beneficial effect of ZCT-, HAP- and KCit-, F- containing toothpaste.

## Conclusions

After 4 weeks, both toothpastes showed good antimicrobial effect with no differences regarding periodontal health. However, the toothpaste containing ZCT, HAP, KCit and F was found to be more effective in reducing the salivary counts of *S. mutans* than the product containing F alone and may also be beneficial for patients experiencing self-reported DH.

## Supplementary information

Below is the link to the electronic supplementary material.

ESM 1 (DOCX 19.2 KB)

ESM 2 (DOCX 21.8 KB)

## Data Availability

No datasets were generated or analysed during the current study.
