# Supplementary material for: The clinical and microbiological efficacy of a zinc-citrate/hydroxyapatite/potassium-citrate containing toothpaste: a double-blind randomized controlled clinical trial
Source: Clin Oral Investig. 2024 Nov 22;28(12):652. doi: 10.1007/s00784-024-06052-z (PMC11582097; doi:10.1007/s00784-024-06052-z)
Supplement: Supplementary file 1 — (DOCX 19.2 KB) [file 784_2024_6052_MOESM1_ESM.docx]

**Pre-enrollment Questionnaire**

Patient Code Number: _________________

1. What toothpaste are you currently using? ______________________________
2. Are your teeth sensitive when you eat or drink?
   - Yes
   - No
3. If yes, how would you rate your dental sensitivity? 1 = “Not sensitive” and 10 = “Very sensitive”

| 1 | 2 | 3 | 4 | 5 | 6 | 7 | 8 | 9 | 10 |
| --- | --- | --- | --- | --- | --- | --- | --- | --- | --- |
| 😊 |  |  |  | 😶 | |  |  |  | ☹️ |

1. How would you rate the taste of the toothpaste? 1 = “Not at all satisfied” and 10 = “Very satisfied”

| 1 | 2 | 3 | 4 | 5 | 6 | 7 | 8 | 9 | 10 |
| --- | --- | --- | --- | --- | --- | --- | --- | --- | --- |
| ☹️ |  |  |  | 😶 | |  |  |  | 😊 |

1. Have you experienced a dry mouth after brushing your teeth?

• Yes

• No

1. In general, how would you rate the toothpaste you are using? 1 = “Not at all satisfied” and 10 = “Very satisfied”

| 1 | 2 | 3 | 4 | 5 | 6 | 7 | 8 | 9 | 10 |
| --- | --- | --- | --- | --- | --- | --- | --- | --- | --- |
| ☹️ |  |  |  | 😶 | |  |  |  | 😊 |

1. Are you satisfied with the current color of your teeth? 1 = “Not at all satisfied” and 10 = “Very satisfied”

| 1 | 2 | 3 | 4 | 5 | 6 | 7 | 8 | 9 | 10 |
| --- | --- | --- | --- | --- | --- | --- | --- | --- | --- |
| ☹️ |  |  |  | 😶 | |  |  |  | 😊 |

**End of the follow-up Questionnaire**

Patient Code Number: _________________

1. Which toothpaste are you currently using (A or B)? ______________________________
2. Are your teeth sensitive when you eat or drink?
   - Yes
   - No
3. If yes, how would you rate your dental sensitivity? 1 = “Not sensitive” and 10 = “Very sensitive”

| 1 | 2 | 3 | 4 | 5 | 6 | 7 | 8 | 9 | 10 |
| --- | --- | --- | --- | --- | --- | --- | --- | --- | --- |
| 😊 |  |  |  | 😶 | |  |  |  | ☹️ |

1. How would you rate the taste of the toothpaste?
   1 = “Not satisfied at all” and 10 = “Very satisfied”

| 1 | 2 | 3 | 4 | 5 | 6 | 7 | 8 | 9 | 10 |
| --- | --- | --- | --- | --- | --- | --- | --- | --- | --- |
| ☹️ |  |  |  | 😶 | |  |  |  | 😊 |

1. Did you experience a dry mouth sensation after brushing your teeth?
   - Yes
   - No
2. In general, how would you rate the toothpaste you are using?
   1 = “Not satisfied at all” and 10 = “Very satisfied”

| 1 | 2 | 3 | 4 | 5 | 6 | 7 | 8 | 9 | 10 |
| --- | --- | --- | --- | --- | --- | --- | --- | --- | --- |
| ☹️ |  |  |  | 😶 | |  |  |  | 😊 |

1. Are you satisfied with the current color of your teeth?
   1 = “Not satisfied at all” and 10 = “Very satisfied”

| 1 | 2 | 3 | 4 | 5 | 6 | 7 | 8 | 9 | 10 |
| --- | --- | --- | --- | --- | --- | --- | --- | --- | --- |
| ☹️ |  |  |  | 😶 | |  |  |  | 😊 |

1. Do you plan to continue using this toothpaste in the future?
   - Yes
   - No
2. Would you recommend it to a friend?
   - Yes
   - No
