# Supplementary material for: The clinical and microbiological efficacy of a zinc-citrate/hydroxyapatite/potassium-citrate containing toothpaste: a double-blind randomized controlled clinical trial
Source: Clin Oral Investig. 2024 Nov 22;28(12):652. doi: 10.1007/s00784-024-06052-z (PMC11582097; doi:10.1007/s00784-024-06052-z)
Supplement: Supplementary file 2 — (DOCX 21.8 KB) [file 784_2024_6052_MOESM2_ESM.docx]

Supplementary Table 1. Clinically assessed parameters at baseline and after 4 weeks of follow-up.

| Variable | Baseline | | 4-week follow-up | | Level of significance |
| --- | --- | --- | --- | --- | --- |
|  | Experimental group | Control group | Experimental group | Control group | p>0.05 |
| Tactile sensitivity  (expressed as % of patients according to Schiff Sensitivity Scale) | 0: 86%  1: 2%  2:10%  3: 2% | 0: 74%  1: 6%  2: 16%  3: 4% | 0: 92%  1: 2%  2: 6%  3: 0% | 0: 81%  1: 5%  2: 10%  3: 4% |  |
| Air-stimilus (expressed as % of patients according to VAS scale) | 0-3: 88%  4-7: 10%  ≥ 8: 2% | 0-3: 84%  4-7:16%  ≥ 8: 0% | 0-3: 92%  4-7: 8%  ≥ 8: 0 | 0-3: 84%  4-7:14%  ≥ 8: 2% |  |
| Plaque index (means ± st.dev) | 0.1 ± 0.5 | 0.17 ± 0.1 | 0.19 ± 0.1 | 0.2 ± 0.1 |  |
| Gingival index | 0 | 0 | 0 | 0 |  |
| Bleeding on probing – Full mouth bleeding score | 0 | 0.21 ± 0.1 | 0.21 | 0.19 ± 0.1 |  |

Supplementary Table 2. Data retrieved from questionnaires at the end of the 4-week follow-up. In case of statistically significant differences between the groups (p<0.05), an asterisk was placed next to the significantly higher value for the outcome.

| Scale-based questions | Experimental group (%) | Control group (%) | Level of significance |
| --- | --- | --- | --- |
| How would you rate your dental sensitivity? 1 = “Barely sensitive”  and 10 = “Very sensitive” | | | |
| Barely sensitive | 22 | 28 | p>0.05 |
| Moderately sensitive | 68 | 49 |  |
| Very sensitive | 10 | 23* | p<0.001 |
| How would you rate the taste of the toothpaste? 1 = “Poorly satisfied  at all” and 10 = “Very satisfied” | | | |
| Poorly satisfied | 10 | 0 | p<0.001 |
| Moderately satisfied | 27 | 7 |  |
| Very satisfied | 63 | 93* |  |
| In general, how would you rate the toothpaste you are using? 1 = “Not satisfied at all” and 10 = “Very satisfied” | | | |
| Not satisfied | 0 | 0 | p<0.001 |
| Moderately satisfied | 64 | 52 |  |
| Very satisfied | 36 | 48* |  |
| Are you satisfied with the current color of your teeth? 1 = “Not satisfied at all” and 10 = “Very satisfied” | | | |
| Not satisfied | 0 | 2 | p>0.05 |
| Moderately satisfied | 30 | 20 |  |
| Very satisfied | 70 | 78 |  |

Supplementary Table 3. Data retrieved from questionnaires at the end of the 4-week follow-up. In case of statistically significant differences between the groups (p<0.05), an asterisk was placed next to the significantly higher value for the outcome.

| Question | Experimental group | | Control group | | Level of significance |
| --- | --- | --- | --- | --- | --- |
|  | Yes  (%) | No  (%) | Yes  (%) | No  (%) |  |
| Are your teeth sensitive when you eat or drink? | 6 | 94 | 36* | 64 | p<0.05 |
| Did you experience a dry mouth sensation after brushing your teeth? | 34* | 66 | 7 | 93 |  |
| Do you plan to continue using this toothpaste in the future? | 41 | 59 | 66* | 34 |  |
| Would you recommend it to a friend? | 60 | 40 | 87* | 13 |  |
